# Nonsense-mediated RNA decay and its bipolar function in cancer

**DOI:** 10.1186/s12943-021-01364-0

**Published:** 2021-04-29

**Authors:** Gonçalo Nogueira, Rafael Fernandes, Juan F. García-Moreno, Luísa Romão

**Affiliations:** 1 Departamento de Genética Humana, Instituto Nacional de Saúde Doutor Ricardo Jorge, 1649-016 Lisbon, Portugal; 2 BioISI – Instituto de Biossistemas e Ciências Integrativas, Faculdade de Ciências, Universidade de Lisboa, 1749-016 Lisbon, Portugal

**Keywords:** Nonsense-mediated RNA decay (NMD), Cancer therapy, Immunotherapy, Neoantigen, Biomarker, Tumor suppressor gene, Oncogene, Environmental stress, Tumor microenvironment

## Abstract

Nonsense-mediated decay (NMD) was first described as a quality-control mechanism that targets and rapidly degrades aberrant mRNAs carrying premature termination codons (PTCs). However, it was found that NMD also degrades a significant number of normal transcripts, thus arising as a mechanism of gene expression regulation. Based on these important functions, NMD regulates several biological processes and is involved in the pathophysiology of a plethora of human genetic diseases, including cancer. The present review aims to discuss the paradoxical, pro- and anti-tumorigenic roles of NMD, and how cancer cells have exploited both functions to potentiate the disease. Considering recent genetic and bioinformatic studies, we also provide a comprehensive overview of the present knowledge of the advantages and disadvantages of different NMD modulation-based approaches in cancer therapy, reflecting on the challenges imposed by the complexity of this disease. Furthermore, we discuss significant advances in the recent years providing new perspectives on the implications of aberrant NMD-escaping frameshifted transcripts in personalized immunotherapy design and predictive biomarker optimization. A better understanding of how NMD differentially impacts tumor cells according to their own genetic identity will certainly allow for the application of novel and more effective personalized treatments in the near future.

## Background

Eukaryotic gene expression comprises a series of interconnected steps in which messenger RNAs (mRNAs) play a crucial intermediate role. To ensure that the genetic information is correctly transcribed from DNA to RNA, and then translated into a functional protein, eukaryotic cells have developed several elaborate quality control mechanisms, many of them acting precisely at the mRNA level [1, 2]. The nonsense-mediated RNA decay (NMD) is one of those mechanisms. NMD recognizes and degrades transcripts harboring a premature translation-termination codon (PTC), preventing the production of C-terminally truncated proteins that can have a deleterious effect in the cell [3, 4]. PTCs can be introduced by mutations in the DNA, such as nonsense or frameshift mutations, or by errors in the mRNA processing [5, 6], and are associated with several diseases, such as β-thalassemia, cystic fibrosis, Duchenne’s muscular dystrophy and cancer [6, 7]. Indeed, it is estimated that 30% of the human genetic diseases are caused by PTC-introducing mutations that, therefore, can be promoted by NMD activity [7]. On other note, transcriptome-wide analyses have revealed that NMD also regulates the abundance of a large number of physiological mRNAs that encode full-length proteins (~ 10% of human wild-type coding genes), suggesting a significant role in gene expression regulation [8–17]. In this regard, NMD function has been implicated in the regulation of many essential biological processes related to organism development, cell differentiation, cell stress and immune responses [18]. Disruption of the NMD mechanism can lead to pathologies, including neurological disorders, immune diseases and cancer.

The involvement of NMD in cancer development has been extensively studied during the past few years. It was shown that different cancer contexts can take advantage of both NMD quality-control and regulatory functions to potentiate the development of the disease [2]. For example, tumor-suppressor genes were shown to be prone to exhibit NMD-inducing features [19], providing an opportunity for NMD to control their expression and potentiate cells sensibility to cancer development [20–25]. On the other hand, NMD was shown to downregulate the expression of many important factors that can aid tumorigenesis, including proteins involved in cell growth, cell cycle, apoptosis and cell migration [26]. In line with this, in some tumors, NMD factors contain disabling mutations that lead to the impairment of NMD-mediated degradation and its protective role against tumorigenesis [27, 28].

The present review describes NMD as a major surveillance and gene expression regulatory pathway, by briefly exposing the knowledge regarding its players, mechanism of action and biological relevance, focusing specifically on cancer. Then, we discuss in detail how tumor cells have explored both the quality and regulatory NMD functions to leverage tumorigenesis in their own microenvironment. We also present the challenges of using NMD in cancer therapy, addressing situations where inhibition or activation of NMD are favorable according to the genetic context of the cancer. Finally, we discuss how NMD inhibition and/or NMD-escaping can contribute for biomarker optimization and personalized immunotherapy design.

## NMD pathway

**Fig. 1:**
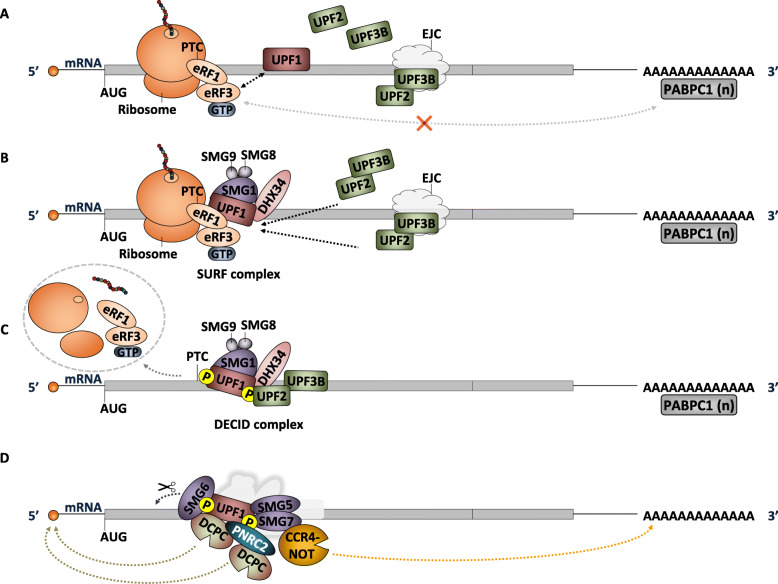
Simplified representation of the nonsense-mediated mRNA decay (NMD) model in mammalian cells. **a** When the ribosome stops at a premature termination codon (PTC), the interaction of UPF1 and eRF3 induces premature translation termination. **b** After this interaction, the SURF complex is formed by eRF1, eRF3, SMG1 associated with SMG8 and SMG9, DHX34 and UPF1. **c** Then, UPF1 interacts with UPF2-UPF3B, either bound to the EJC downstream of the PTC (EJC-dependent NMD model) or diffused in the cytoplasm (EJC-independent NMD model), to form the DECID complex and induces the SMG1-mediated phosphorylation of UPF1. At this point, translation has terminated with the dissociation of the ribosomal subunits, the release factors and the nascent peptide. **d** Phosphorylated UPF1 triggers the decay phase by recruiting factors that lead to mRNA degradation, such as SMG6, which produces an endonucleolytic cleavage, SMG5-SMG7 dimer, which recruits the CCR4-NOT deadenylase complex, and/or PNRC2, which recruits the decapping complex (DCPC)

The molecular mechanism of NMD has been described by several models that differ in some key aspects, while agreeing that it is fundamentally a translation-dependent process. This dependence has been observed over the years using translation inhibition approaches, either by the presence of translation inhibitors (e.g., cycloheximide and puromycin) or extended stem-loops in the 5′ untranslated region (UTR) [29, 30]. Currently, there are two main models that better describe the NMD mechanism: the exon junction complex (EJC)-dependent and the EJC-independent model (Fig. 1) [31], which differ mostly in the involvement of an EJC, a dynamic multiprotein complex, that includes up-frameshift protein 3B (UPF3B), an important NMD-factor [32–35]. The EJC is formed during splicing and deposited 20-24 nucleotides (nts) upstream of exon-exon junctions, remaining associated with the mRNA during its transport to the cytoplasm until its displacement by the translating ribosome during the pioneer round of translation [32–34, 36, 37]. In a situation where an mRNA contains a PTC located more than 50–54 nts upstream of the last exon-exon junction, the ribosome is unable to displace the downstream EJCs [38, 39], allowing an interaction between the latter and certain NMD factors, thus triggering NMD [38, 40–42].

NMD begins with the arrival of the translating ribosome to a PTC, at which the translation eukaryotic release factors (eRF) 1 and 3 interact with UPF1 and the serine/threonine protein kinase, SMG1, associated with its regulators, SMG8 and SMG9, to form the “SURF” complex (Fig. 1b) [42–44]. Recent evidence places the ATP-dependent RNA helicase DEAH box polypeptide 34 (DHX34) in this complex, operating as a scaffold protein for UPF1-SMG1 interaction [45, 46]. Meanwhile, UPF3B and UPF2 are recruited to interact with UPF1 [45, 46], forming the decay-inducing complex (DECID) (Fig. 1c) [44, 47]. The interaction between UPF1, UPF2 and UPF3B leads to conformational changes in UPF1 structure allowing the subsequent SMG1-mediated UPF1 phosphorylation and the exit of the ribosome, the nascent peptide and the release factors (Fig. 1c) [42, 44, 47–53]. For NMD activation to occur, the EJC-independent model depends on the diffusion of UPF2 and UPF3B in the cytoplasm, while the EJC-dependent model relies on their interaction with the EJC downstream of the PTC (Fig. 1a) [31]. However, not all PTC-containing transcripts are able to induce NMD. For example, when the cytoplasmic poly(A)-binding protein 1 (PABPC1) bound to the poly(A) tail is near the termination complex at the PTC, it can repress NMD by interacting with eRF3 and preventing the UPF1-eRF3 interaction [54–57]. The PABPC1-eRF3 interaction is thought to be essential for normal termination [42, 58, 59]. This competition appears to be more relevant in the EJC-independent NMD mechanism since it relies on UPF2 and UPF3B diffusion [54–57, 60].

The steps following UPF1 phosphorylation are common to both NMD models. Once phosphorylated, UPF1 triggers mRNA decay. First, by taking advantage of its RNA helicase activity to remove the RNA secondary structures and proteins downstream of PTCs [61]. Second, through the recruitment of SMG6, SMG5-7 heterodimer, or SMG5 and PNRC2 (Proline Rich Nuclear Receptor Coactivator 2) (Fig. 1d) [52, 62], causing the dissociation of SMG1, SMG8 and SMG9. Additionally, the protein phosphatase 2A (PP2A) dephosphorylates UPF1. The endonuclease activity of SMG6 induces cleavage in the vicinity of the PTC, generating unprotected mRNA ends that will be prone to degradation [63–67]. When SMG5-SMG7 or SMG5-PNRC2 are present, these proteins further recruit the decapping complex (DCPC) and the deadenylation complex (CCR4-NOT) to remove the cap-binding complex and the poly(A) tail (Fig. 1d), allowing 5′-to-3′ and 3′-to-5′ RNA degradation by XRN1 and the RNA exosome, respectively [63, 64, 68–71].

The formation of UPF1-UPF2-UPF3 surveillance complex is believed to activate NMD, however, recent studies by Neu-Yilik et al., presented the possibility that UPF3B, rather than UPF1, interacts with eRF3 to induce peptide release and dissociation of the termination complexes. Moreover, UPF3B was also shown to interact with UPF1, contradicting the need for UPF2 to bring together these two NMD factors [72]. Such findings propose a novel role for UPF3B that requires further testing [73]. In sum, the proper mRNA targeting and decay by the NMD pathway depends on the presence of several proteins in a spatiotemporal manner, as discussed above. Hence, one can expect the existence of less common branches of NMD linked to the regulation of specific targets. In fact, it was shown in higher eukaryotes that, besides de EJC-dependent and independent branches, NMD could also be UPF2-independent and UPF3-independent [34, 74, 75]. In support of this view, Huang et al. found that NMD owns an auto-regulatory feedback loop mechanism that is dependent on the different branches of NMD: the UPF3B-dependent branch regulates UPF1 and SMG7 transcripts, the EJC-dependent branch regulates UPF1 and SMG5 transcripts, and the UPF2-dependent branch regulates SMG1 mRNA levels [31, 76]. Furthermore, it was found that a splicing factor, SRSF1 (Serine and Arginine Rich Splicing Factor 1), directly interacts with UPF1, enhancing the binding of the latter to the mRNA, thus promoting NMD. Since this event is both UPF3B and UPF2-independent, it might constitute a new NMD branch [77]. In the human organism, it is not known if the efficiency of these NMD branches varies between different tissues.

As mentioned before, the NMD pathway was initially classified as a quality control mechanism responsible for the targeted degradation of aberrant transcripts carrying a PTC. However, during the last decade, several transcriptome-wide studies revealed that NMD also degrades a variety of normal mRNAs that would produce functional proteins. This role classifies NMD as major regulatory mechanism of gene expression, being directly or indirectly responsible for the regulation of ~ 3–20% of transcripts in eukaryotes from yeast to mammals [8, 9, 11, 13–16, 34, 78, 79]. In an attempt to elucidate why NMD targets these apparently normal transcripts, several studies have reported the existence of NMD-inducing features that are responsible for eliciting their decay. For instance, mRNAs with long 3’UTRs have higher probability of eliciting NMD, due to PABPC1 failing to interact with the termination complex [57, 80–82]. Another NMD-triggering feature is the presence of at least one exon-exon junction more than 50 nts downstream of the termination codon [83]. An additional feature is the presence of upstream open reading frames (uORFs) in the 5′UTR of the transcripts [84]. In this case, the NMD is triggered by the stop codon of the uORF which is at the 5′-end of the mRNA with downstream EJCs, thus placing it in a premature context [84]. These genomic features are considered the three canonical rules of NMD targeting, yet it is possible for a transcript that carries one or more of these genomic features to evade NMD. The variance in NMD efficiency across thousands of PTCs still requires further elucidation. More recently, based on data from paired cancer exomes and transcriptomes, Lindeboom et al. proposed two additional non-canonical features able to trigger NMD: the long exon rule and the start-proximal rule [21]. In this study, the authors propose that PTCs located in exceptionally long exons (> 400 nts) significantly reduce NMD efficiency and that PTCs at < 150 nts from the start codon in most cases fail to induce NMD, probably due to translation re-initiation at a downstream AUG codon [21, 85]. In addition, the same authors also observed that PTCs located very far from the normal stop codon induce a low efficient NMD process, which they called “the PTC-to-normal-stop rule” [21, 85]. Furthermore, they found that mRNAs with short half-lives are targets of an inefficient NMD [21, 85]. Still, the same work has shown that the presence of specific *cis*-acting elements in the mRNA, which can be located either near to the PTC or in the natural UTR of the transcript, can modulate NMD efficiency [21, 85]. These data demonstrate that there are many different genomic features involved in targeting a transcript for NMD. In fact, when certain criteria are met, NMD is able to regulate the levels of a subset of normal transcripts, linking its activity to the regulation of many biological pathways.

## NMD biological relevance

Considering the NMD dual function, one can expect its activity and regulation to be involved in both physiological and pathophysiological mechanisms. In fact, NMD activity has been implicated in several biological processes related to cell differentiation, cell stress and immune responses [18]. Also, NMD presents a special role in modulating human diseases caused by PTC-introducing mutations, as the ones observed in β-thalassemia, cystic fibrosis, Duchenne’s muscular dystrophy and cancer [6, 7].

### NMD modulates biological processes

Over the years, a strong correlation between depletion of core NMD factors and embryonic lethality has been established [18, 86–89]. Accordingly, NMD-mediated regulation of two pro-apoptotic factors, GADD45 (Growth Arrest and DNA Damage 45) and GAS5 (Growth Arrest Specific 5), is important to maintain mammalian cell viability and development [90–92]. Moreover, depletion of NMD factors such as UPF2 and UPF3 can, respectively, lead to: defective liver development and regeneration [93], and dysregulation of genes essential for spermatogenesis, causing severe testicular atrophy and male sterility [18, 94–96]. Although NMD seems to play a role in the regulation of these processes, we cannot exclude the possibility that other mechanisms are operating and contributing to the phenotype [97, 98]. An example occurs in myogenesis, where the competition between NMD and STAU-mediated decay (SMD) for the exclusive interaction with UPF1 was shown to be essential [97, 98]. During differentiation from myoblasts to myotubes NMD activity decreases to upregulate the promyogenic transcription factor, myogenin, while SMD efficiency increases, downregulating the antimyogenic factor PAX3 (Paired Box Gene 3) [97, 98].

NMD has also been implicated in the proliferation/differentiation of cells during brain development [99]. If stimulated, NMD inhibits neural differentiation and sustains a proliferative state [99]. Accordingly, it was shown that during neural development, NMD activity is downregulated by microRNAs to allow upregulation of transcripts encoding proteins important for neural differentiation and maturation processes [100]. Mutations in NMD factors can lead to dysregulation of genes related to neural function, implicating NMD in neural pathologies such as X-linked intellectual disability, schizophrenia and autism [10, 101–103].

Several studies have also linked NMD function to different stress conditions, such as amino acid starvation, hypoxia, reactive oxygen species (ROS) and ER stress [14, 26, 104–108]. In response to different stress stimuli, specific kinases are activated to phosphorylate the eukaryotic initiation factor-2α (eIF2α) [107]. Phosphorylation of eIF2α affects its interaction with the GTP-GDP exchange factor eIF2B, resulting in low availability of the eIF2 ternary complex (eIF2-GTP-tRNAi) which causes a general reduction in protein synthesis, and thus, in NMD activity [8, 105, 106, 108–112]. Alternatively, data suggests that eIF2α phosphorylation also promotes formation of cytoplasmic stress granules and relocalization of NMD components into these granules, resulting in a spatial separation between untranslated mRNAs and the RNA degradation machinery that represses NMD activity [8, 105, 106, 108–111]. Accordingly, it was shown that numerous stress-related mRNAs are targeted by NMD under basal conditions, and that during stress, NMD inactivation promotes their upregulation to solve the stress [8, 110–112]. Among these targets are important factors of the stress-response pathways, the integrated stress response (ISR) and the unfolded protein response (UPR), such as the transcription factors, ATF4 (Activating Transcription Factor 4), ATF3 (Activating Transcription Factor 3) and CHOP (CCAAT-Enhancer-Binding Protein Homologous Protein) [105, 106, 109, 113]. Here, we discussed a possible mechanism by which stress responses impact NMD, but it is important to know if other mechanisms are also involved. Furthermore, it could be interesting to explore these mechanisms in order to develop novel therapies for those diseases associated with cellular stress conditions, including cancer.

NMD has also been shown to play a relevant role in viral replication, since the abrogation of this pathway culminates in the upregulation of viral proteins and subsequent higher viral infection, indicating that NMD plays a host protective role in blocking virus replication, such as the Semliki Forest virus (SFV) and Sindbis virus (SINV) [114]. In response, some viruses have evolved mechanisms to evade or inhibit NMD: the Rous sarcoma virus has a stability element (RSE) in its 3’UTR that protects the viral RNA, and the HTLV-1 virus expresses two proteins, Tax and Rex, that inhibit NMD [115, 116].

**Fig. 2:**
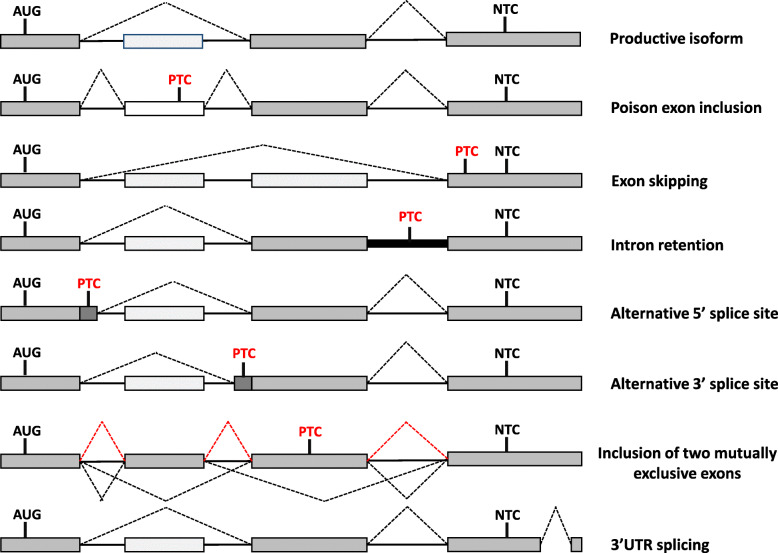
Schematic representation of alternative splicing (AS) patterns inducing nonsense-mediated mRNA decay (NMD)-sensitive mRNA isoforms. The first mRNA isoform represents a productive transcript that encodes a functional protein. The following isoforms represent NMD-sensitive transcripts promoted by different AS patterns. Poison cassette inclusion leads to the retention of a premature translation-termination codon (PTC)-containing exon, while exon skipping can induce a frameshift in the mRNA sequence, introducing a downstream PTC. On the other hand, intron retention and alternative 5′/3′ splice sites may promote an NMD-target by including an in-frame PTC. Inclusion of two exons that usually are spliced separately may result in a frameshift, creating a PTC-positive isoform. The last isoform represents an AS pattern that leads to skipping in the 3′ untranslated region (3’UTR), which results in the presence of an exon junction complex downstream of the normal termination codon, favoring a premature context. AUG: start codon; NTC: normal termination codon

The physiological relevance of NMD is further expanded by its connection with alternative splicing (AS) [117, 118]. AS is the greatest contributor to proteome diversity in the cell by generating distinct mRNA isoforms from a single pre-mRNA [119–121]. AS is also the molecular mechanism that generates most of the natural NMD-targets, being estimated that around one third of all AS events results in PTC-containing transcripts [122, 123]. The coupling of AS to NMD (AS-NMD) constitutes a conserved post-transcriptional mechanism of gene expression regulation, that balances the ratio of productive and unproductive mRNA isoforms [117]. Ultimately, this coordinated process leads to regulated protein synthesis, by sorting which of the already transcribed pre-mRNAs will end in a mature mRNA proper for translation. Several AS patterns render NMD-sensitive mRNA isoforms, like exon inclusion and skipping, intron retention in the 3’UTR, or alternative usage of 5′ or 3′ splice sites (Fig. 2) [124, 125].

Splicing factors are key mediators of AS-NMD by shifting the balance of which splice sites are recognized, in order to promote transcripts bearing in-frame nonsense codons, or PTC-free isoforms [117]. Interestingly, several splicing factors use AS-NMD to regulate their own expression through autoregulatory negative feedback loops by binding to their own transcripts and promoting the production of NMD-sensitive isoforms [126–134]. This phenomenon has been described for numerous members of the serine arginine-rich (SR) proteins and heterogeneous nuclear ribonucleoproteins (hnRNPs) [126–130]. Frequently, serine and arginine-rich (SR) proteins act as splicing activators [131, 132], as observed for SRSF2 which targets its own transcript to favor the inclusion of an exon bearing a PTC, hence yielding an NMD-sensitive isoform [126]. In contrast, most hnRNPs function as splicing repressors, promoting exon skipping, as reported for the polypyrimidine tract-binding protein 1 (PTBP1) [129, 135]. Besides these splicing factor families, autoregulation by AS-NMD has been also observed in other RBPs, like core spliceosomal components and ribosomal proteins [133–135]. Altogether, these data constitute strong evidence that AS-NMD is a determinant post-transcriptional mechanism that is essential to control gene expression and strongly impacting the transcriptome.

### NMD modulates disease phenotype

As NMD has a key role in mRNA surveillance, it is not surprising that its activity has been implicated in the phenotype modulation of several human genetic diseases [6, 7]. For instance, the β-thalassemia, a well-known inherited severe anemia caused by low levels of normally structured β-globin, can be induced by the presence of nonsense mutations in the β-globin gene [6, 7]. In this situation, the mutated allele is recognized and degraded by NMD, thus preventing the formation of a truncated peptide product with potentially toxic effects to the cell [6, 7]. This way, a heterozygous carrier of such mutation can still depend on the normally functioning wild type allele. However, there are cases where the PTC context does not trigger NMD leading to a dominant phenotype [6, 7]. Other examples of this relationship include susceptibility to mycobacterial infection [136, 137], von Willebrand disease [138], factor X deficiency [139], retinitis pigmentosa and blindness [140, 141] and pseudoxanthoma elasticum [142]. In contrast, there are genetic disorders where truncated peptides may retain function and induce mild disease phenotypes. However, in these disorders, the NMD pathway acts to worsen the disease phenotype by degrading NMD-sensitive transcripts that would express partially-functional proteins. This type of phenotype modulation has been reported in diseases such as Duchenne muscular dystrophy, cystic fibrosis [143, 144], and spinal muscular atrophy [145].

While in most of the above-mentioned genetic diseases the quality-control facet of NMD is particularly relevant, different aspects of NMD activity have been explored by cancer cells to potentiate the disease [139]. Indeed, in normal circumstances, NMD has a protective role against cancer [26], acting as a tumor suppressor pathway through the downregulation of proteins involved in cell growth, cell cycle, apoptosis and cell migration [26]. However, some studies reported that tumor cells found ways to take advantage of NMD functions to potentiate the development of the disease [2]. In some cases, tumors may benefit from NMD-mediated degradative activity by selecting the acquisition of PTCs in tumor-suppressor genes that allow the growth of tumor cells [20–25]. Other tumors arise from NMD impairment either by mutations in NMD factors or its stress-mediated inhibition [2, 26–28, 107, 146–150]. In the following sections we will discuss deeper the complexity of this relation between NMD and cancer.

## The complex role of NMD in cancer

Tumors have found ways to leverage their unconstrained growth and the progression of cancer by taking advantage of both functions of NMD. On one hand, cancer cells use NMD activity to selectively downregulate tumor-suppressive genes through PTC acquisition, but on the other hand, these cells fine-tune NMD magnitude to allow the upregulation of stress-corrective genes responsible for their adaptation to the tumor microenvironment [21, 26]. Although apparently straightforward, the role of NMD in cancer development and progression can, in fact, be quite complex [2, 26, 148, 149]. While in some contexts NMD may work as a tumor-suppressive pathway, in other scenarios its activity might aggravate the disease, depending on the genetic evolutionary history of the tumor [2, 151].

### NMD activity against tumorigenesis

**Fig. 3:**
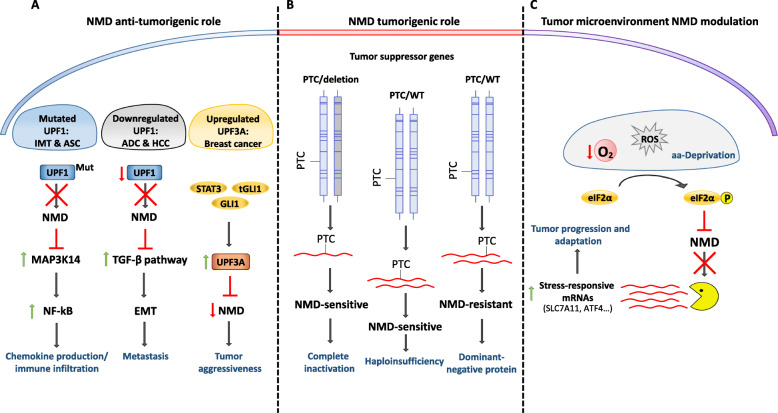
Roles of nonsense-mediated mRNA decay (NMD) in cancer. **a** Disabling mutations or changes in the gene expression level of the key NMD factors (UPF1 as an example) occur in different cancer types [for example, lung inflammatory myofibroblastic tumor (IMT), pancreatic adenosquamous carcinoma (ASC), lung adenocarcinoma (ADC), and hepatocellular carcinoma (HCC)]. The case on the left represents mutated (Mut) UPF1, which leads to a decreased NMD activity resulting in the upregulation of an NMD target encoding the mitogen activated protein kinase kinase kinase 14 (MAP 3 K14) and stimulating NF-κB (nuclear factor kappa-light-chain-enhancer of activated B cells) activity, thus inducing chemokine production and immune infiltrations. The example in the middle illustrates lower NMD activity due to the downregulation of UPF1, which causes the upregulation of several factors of the transforming growth factor beta (TGF-β) pathway. This favors the epithelial-mesenchymal transition (EMT) and consequently, the number of metastatic events. The example on the right shows the interaction between three oncoproteins, STAT3, GLI1 and tGL1, to induce higher protein levels of UPF3A, which inhibits NMD activity, increasing malignant progression of the tumor. **b** Tumor suppressor genes can completely loss their function by PTC acquisition and subsequent NMD degradation, combined with either deletion of the wild-type allele, or haploinsufficiency of the remaining allele. On the other hand, a tumor suppressor gene can experience an NMD-resistant mutation, leading to a dominant-negative protein that hampers the wild-type function. **c** The tumor microenvironment modulates NMD in order to overcome different types of cellular stresses associated with the unconstrained growth of the tumor. Stresses such as hypoxia, production of reactive oxygen species (ROS), or nutrient deprivation promote, eIF2α phosphorylation, which inhibits NMD and therefore, several mRNAs encoding stress-responsive factors are stabilized, allowing tumor progression and adaptation. WT: wild type; PTC: premature termination codon; aa: amino acid

Disabling mutations in genes encoding NMD factors have been found in several types of cancers, which raised the possibility of NMD having some type of protective role against tumorigenesis. For instance, in pancreatic adenosquamous carcinoma (ASC) tumors and in lung inflammatory myofibroblastic tumors (IMTs), UPF1 gene exhibits splicing-altering mutations that compromise NMD activity [27, 28]. This may lead to the upregulation of genes typically controlled by NMD that, therefore, contribute for the disease phenotype. For example, the NMD target encoding the mitogen activated protein kinase kinase kinase 14 (MAP 3 K14 or NIK), a potent activator of the proinflammatory nuclear factor-kappa B (NF-κB) signaling pathway, is upregulated in the UPF1-mutated IMTs, promoting chemokine production and immune infiltrations that characterize this type of tumors [28]. Similarly, lung adenocarcinomas (ADCs) and hepatocellular carcinomas (HCCs) display lower expression of UPF1 when compared to normal tissues due to promoter hypermethylation, a finding that correlates with poor prognosis in patients with HCC [147, 152]. The resultant impairment of the NMD pathway leads to the upregulation of factors from the transforming growth factor beta (TGF-β) signaling pathway, which drive epithelial-mesenchymal transition (EMT) and, consequently, tumorigenesis and neoplasm metastasis [147]. In line with these clinical findings, there is experimental data showing that overexpression of UPF1 reduces the number and size of cultured tumor cell colonies when compared to control cells of several cancer cell lines [26]. Also, UPF1-overexpressing prostate cancer (PC3) cells injected as tumor explants in nude mice present no significant tumor growth [26]. Interestingly, an expression array analysis performed in human bone osteosarcoma (U2OS) cells subjected to NMD inhibition revealed that NMD controls the expression of a wide variety of transcripts that encode important factors involved in tumorigenesis-related processes, including cell growth, cell cycle, growth factor signaling, apoptosis and cell migration [26]. Altogether, it seems that typically NMD works as a tumor suppressor pathway by regulating the expression of genes involved in cell proliferation, differentiation and survival, and that tumors with impaired NMD, like the ones with mutated UPF1, have favorable conditions for tumor proliferation (Fig. 3a). Further supporting this idea is a study reporting that UPF3A, a paralog of UPF3B that inhibits NMD by sequestering UPF2 [47], is highly expressed in metastatic tissues of colorectal cancer (CRC) when compared to primary tissues [153]. This higher expression is associated with liver metastasis, recurrence and poor prognosis in CRC patients [153]. This finding was also reported in a previous study in which the interaction of the oncogenic transcription factors, signal transducer and activator of transcription 3 (STAT3), glioma oncogene homolog 1 (GLI1), and truncated GLI1 (tGLI1), was seen to promote UPF3A upregulation [154]. That higher UPF3A expression enhances the aggressiveness of triple-negative and human epidermal growth factor receptor 2 (HER2)-enriched breast cancers and worsens metastasis free survival, according to the gene expression profile of breast tumors retrieved from the GEO database [154]. Thus, it is possible that some tumors with fully functional NMD factors may have found another way to control NMD activity by increasing the expression of modulators, like UPF3A, to leverage tumorigenesis and/or drive the disease (Fig. 3a).

In addition to its role in gene expression regulation, NMD ability to degrade PTC-harboring transcripts may also be involved in the protection of cancer development. It was shown that tumor suppressor genes have a higher propensity to acquire nonsense mutations than oncogenes, which present mostly missense mutations [19]. Moreover, many of these nonsense mutations are predicted to induce NMD [19]. Indeed, several tumor suppressor genes were found to present PTC-introducing mutations in a plethora of cancers, like p53 in mantle-cell lymphoma [23] and breast cancers [20], E-cadherin (CDH1) gene in hereditary diffuse gastric cancers (HDGC) [155], retinoblastoma 1 (RB1) gene in mantle-cell lymphoma [23], breast cancer type 1 susceptibility protein (BRCA1) gene in breast and ovarian cancers [22], and breast cancer type 2 susceptibility protein (BRCA2) gene in breast cancers [25]. Interestingly, the abnormal transcripts that result from these mutated tumor suppressor genes are stabilized in conditions of inhibited NMD, suggesting that this pathway is usually responsible for degrading them [20, 22, 23, 25, 155]. Therefore, in these contexts, the quality-control function of NMD may protect the cell from production of potential dominant-negative proteins that would, otherwise, lead to tumorigenesis, as has been described in different models, like BRCA1 in nude mice [156], p53 in human samples [27], or the Wilms’ tumor protein 1 (WT1) gene in in vitro studies [24].

### NMD activity in cancer development and progression

Despite the clear notion that NMD can work against cancer, in some contexts its activity may have the opposite effect. For instance, it was recently reported that NMD inhibition in CRCs with microsatellite instability (MSI) leads to decreased tumor growth in xenograft models, suggesting that NMD typically plays a pro-tumorigenic role in these tumors [157]. Indeed, CRCs with MSI present a higher expression of UPF1, UPF2, SMG1, SMG6, and SMG7, when compared to the counterparts CRCs with microsatellite stability. The overexpression of NMD factors is thought to potentiate degradation of the increased number of potentially toxic PTC-bearing transcripts that MSI CRCs characteristically produce, promoting their survival [157]. In a different context, if a PTC-harboring tumor suppressor gene encodes a truncated protein that totally- or partially- preserves its original function, rather than a dominant-negative one like described above, the targeted degradation of its mRNA by NMD could promote cancer development. Accordingly, patients with NMD-sensitive mutations in CDH1 present a higher risk of developing HDGC than patients with NMD-resistant mutations, possibly because the latter produce truncated but still functional forms of E-cadherin [155]. Interestingly, in a study matching exome and transcriptome data from human tumors it was reported that nonsense mutations are enriched in regions of tumor suppressor genes expected to induce NMD [21]. As these mutations usually occur in heterozygosity [19], this raises the question of how NMD potentiates cancer development when at least one allele of a tumor suppressor is functional [158]. Regarding this, Lindeboom et al. have revealed that cancer cells take advantage of NMD activity to accomplish complete tumor suppressor inactivation, which can occur by three mechanisms: i) tumor selection for NMD-inducing mutations in one allele combined with a deletion in the second allele, thus achieving biallelic inactivation by a “two-hit” process; ii) selection for NMD-inducing mutations in haploinsufficient versions of the wild-type allele, to eliminate residual function; and less frequently, iii) selection for NMD-resistant mutations in alleles that produce dominant-negative proteins [21] (Fig. 3b).

In addition to participating in the process of cancer development, NMD activity also seems to impact tumor evolution. During cancer progression, tumor cells acquire several somatic mutations that may, or not, favor the tumorigenic process. This is accompanied by a positive and negative selection that allows the proliferation of subclones with favorable mutations, such as PTCs in tumor suppressor genes or NMD-resistant/missense mutations in oncogenes, while eliminating the ones bearing detrimental mutations. By this means, cancer cells take advantage of NMD activity and of the rules that govern its induction to favor proliferation of transformed cells overproducing oncoproteins and other pro-tumorigenic proteins, thus driving further and aggravating the disease [19, 21].

#### A role for AS-NMD in cancer

As explained before, NMD activity is intimately linked to AS, together forming a key post-transcriptional mechanism of gene expression regulation. During the last decade, many dysregulated AS events have been observed in several cancer types, typically involving mutated splicing factors that lead to genome-wide altered patterns of gene expression. This can result in the production of NMD-sensitive isoforms of oncogenes and tumor suppressor genes that will, therefore, contribute for cancer development. A well-documented example is the SR protein, SRSF2, frequently mutated in patients with acute myeloid leukemia (AML) [159, 160]. A recent study reported that Pro95 hot spot mutation in SRSF2 enhances the stabilization of EJCs downstream from the PTC, thus favoring the association of key NMD factors to elicit NMD [159]. A robust target of SRSF2Mut is EZH2, which encodes a protein that catalyzes histone methylation and acts as tumor suppressor in myeloid malignancies [161]. SRSF2Mut drives the inclusion of a poison exon in the EZH2 pre-mRNA that triggers NMD and consequently shuts down its protein expression [159]. In agreement with this finding, there are studies reporting EZH2 loss-of function mutations in the same spectrum of myeloid disorders displaying mutated SRSF2 [162, 163]. These data suggest that Pro95 mutation turns SRSF2 into an oncoprotein that uses AS-NMD to shut down the expression of a tumor-suppressor gene.

Epithelial cadherin (E-cadherin) is a crucial factor to maintain tissue integrity and polarization of epithelial cell layers and it is well-known that E-cadherin loss is a key event during cancer progression that contributes to the epithelial-mesenchymal transition [164]. Interestingly, Matos et al. discovered that one of the causes leading to E-cadherin decrease is an mRNA variant produced by AS that is committed to NMD [165]. That novel isoform arises from the usage of an alternative 3′ splice site that ends in the depletion of 34 nucleotides in the exon 14, introducing a PTC. Moreover, stable breast cancer MCF7 cells expressing this novel variant resulted in a concomitant decrease of the wild type E-cadherin mRNA levels and higher cell migration and invasiveness [165]. However, it has to be elucidated the mechanism by which AS promotes this alternative isoform. Another example of an AS-NMD event impacting EMT is the one orchestrated by SRSF1. This splicing factor promotes a constitutively active isoform of the proto-oncogene MST1R ((Macrophage Stimulating 1 Receptor), by inducing skipping of exon 11 [166]. Consequently, the active isoform of MST1R induces EMT, as well as increases resistance to apoptosis [167–169]. AS-NMD operates upstream in this pathway, inducing 3’UTR intron retention in SRSF1 under physiological conditions, which creates a stop codon premature context inducing NMD. However, in a tumorigenic context, another splicing factor, KHDRBS1 (KH RNA Binding Domain Containing, Signal Transduction Associated 1), stabilizes SRSF1 mRNA, which turns into a positive regulation of constitutive active MST1R.

Hypoxia is a major feature in solid tumors, given the high proliferating mass of cancer cells encountering an avascular environment that limits oxygen supply [170]. Interestingly, hypoxia seems to impact AS-NMD, as observed for the cysteine-rich angiogenic inducer 61 (CYR61), a matricellular protein that promotes cell proliferation, migration and angiogenesis in numerous solid tumors [171–173]. Under normal conditions, CYR61 experiences retention of intron 3, which translates into a downstream PTC, leading to an NMD-sensitive isoform [174]. Nevertheless, hypoxic conditions alter this AS-NMD gene regulation, inducing skipping of intron 3, which makes the transcript resistant to degradation by NMD.

N^6^-Methyladenosine (m^6^A) RNA methylation is a common mRNA modification dynamically regulated in mammalian cells [175] that controls several steps of the mRNA metabolism, from mRNA processing and transport to translation or decay [176–179]. METTL3, the catalytic subunit of the m^6^A methyltransferase complex, plays a critical role on tumorigenesis, promoting cell proliferation, survival, and invasion of cancer cells [180–182]. Interestingly, Li et al. recently discovered that METTL3 modulates alternative splicing of splicing factors affecting the pool of PTC-bearing isoforms in glioblastoma cells [183]. Transcriptome studies showed that impaired METTL3 methylation results in the generation of PTCs in the mRNAs of several SRSFs, contrary to the existing scenario in malignant gliomas, which are associated with elevated expression of METTL3 and lower expression levels of SRSFs NMD spliced forms [183]. Moreover, authors demonstrated that oncogenicity derived from METTL3 activity is due to changes in alternative splicing events of genes with relevant implications in cancer cell death and migration, like *BCL-X* and *NCOR2*. Altogether, this clearly indicates that dysregulated AS events in cancer cells contribute for the involvement of NMD in cancer development and/or progression.

### NMD modulation in the tumor microenvironment

In striking contrast with the previous examples in which NMD promotes cancer is the finding that the tumor microenvironment induces NMD attenuation to potentiate cancer progression and adaptation, stressing out the idea that NMD activity has paradoxical outcomes. As mentioned above, during the unconstrained growth of the tumor, the blood supply to cancer cells becomes insufficient, creating a cellular environment of hypoxia, nutrient deprivation, production of ROS and ER stress, all stimuli that promote phosphorylation of eIF2α and inhibit NMD [26, 105, 107, 109, 148] (Fig. 3c). Accordingly, it has been reported that cancer cells grown as three-dimensional tumor explants present decreased NMD activity when compared to cancer cells cultured in monolayers, displaying significant levels of eIF2α-P [26]. In fact, several studies showed increased levels of eIF2α-P in a plethora of cancers, including bronchioloalveolar, gastrointestinal and thyroid carcinomas, Hodgkin’s lymphoma, melanocytic and colonic epithelial neoplasms, and breast cancer [184–189]. The downside effect of this stress-mediated NMD inhibition in tumors is the stabilization and upregulation of several transcripts encoding stress-responsive factors, which will help cancer cells to proliferate in the adverse environment of the tumor [26] (Fig. 3c). For instance, it has been reported that NMD inhibition by stress stabilizes the mRNA of the cystine/glutamate antiporter xCT [Solute Carrier Family 7 Member 11 (SLC7A11)], a subunit of the xCT amino acid transporter system. This rate-limiting channel is responsible for the uptake of cystine for production of the cellular antioxidant, glutathione (GSH), a tripeptide that neutralizes free radicals and reactive oxygen compounds. Curiously, it was shown that UPF1-depleted cells can survive to higher doses of H2O2 in a SLC7A11-dependent manner, suggesting that NMD impairment during stress, and the consequent upregulation of SLC7A11, provides protection to cancer cells against the oxidative damage that may result from the overproduction of ROS [106]. The ATF4 mRNA is another NMD target frequently found to be upregulated in several types of solid tumors, consistent with NMD inhibition in these conditions [190, 191]. Nutrient deprivation and oxidative stress promote ATF4 expression to transcriptionally induce genes involved in amino acid synthesis and transport, protein folding, cell differentiation, and autophagy, as a way to counterbalance the stress. Interestingly, it has been reported that the simultaneous molecular/pharmacological inhibition of autophagy and NMD leads to synergistic cell death in CRC cell lines, suggesting that autophagy is an adaptive response to NMD inhibition that allows cancer cells to overtake metabolic stress [192]. Moreover, ATF4 activity has been implicated in the chemo-resistance of CRC cells [190], further highlighting that NMD shut down by the tumor microenvironment can favor signaling cascades that potentiate tumor proliferation and malignancy. Altogether, these findings indicate that NMD impairment is a consequence and part of the adaptative mechanisms cancer cells use to thrive in the harmful microenvironment of the tumor.

## Using NMD modulation in cancer therapy

The fact that NMD can both act as a tumor suppressor and a tumor promoter pathway depending on the genetic context of the cancer, imposes a challenge when it comes to using it in cancer therapy. NMD inhibition therapy can favor patients with tumors displaying PTCs in tumor suppressor genes encoding partially or fully functional proteins. However, this is not the case of tumors displaying a stress-adaptative gene expression pattern, in which NMD activation therapy would be more adequate. Nevertheless, global or gene-specific NMD modulation has great potential to be used in cancer therapy, being intensively studied with some very promising results.

### NMD activation in cancer therapy

**Fig. 4:**
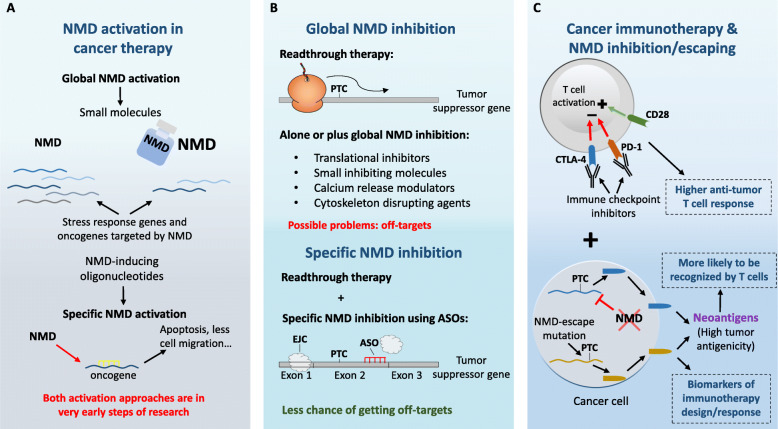
Summary of nonsense-mediated mRNA decay (NMD) inhibition/escaping and activation strategies for cancer treatment. **a** NMD activation in cancer therapy. The search of small molecules boosting NMD activity is still under research, but supposes a potential treatment for those cancer types where tumor-suppressive functions of NMD are beneficial. A more developed and gene-specific approach is the use of oligonucleotides designed to promote NMD over cancer- and/or stress-related transcripts. **b** Global and gene-specific NMD inhibition in cancer therapy. Nonsense suppressor compounds inducing read-through of premature termination codons (PTCs) by the ribosome allow the synthesis of full-length tumor suppressor proteins. This approach can be combined with global NMD inhibition to increase steady-state PTC-containing transcript levels by the use of translational inhibitors, small inhibiting molecules, calcium release modulators or cytoskeleton disrupting agents. In order to avoid non-desired off-targets affected by the general NMD inhibition, antisense oligonucleotides (ASOs) blocking the deposition of an EJC downstream of a PTC allows gene-specific targeting. EJC: exon junction complex. **c** Global NMD inhibition in cancer immunotherapy. Immune checkpoint inhibitors against negative regulators of T cell activation like CTLA-4 or PD-1 are commonly used in cancer immunotherapy to boost the anti-tumor immune response. NMD inhibition is a potential adjuvant therapy given the propensity of the tumor to produce neoantigens that trigger the immune response. Cancer cells experience numerous frameshift mutations resulting in PTC-containing transcripts that upon NMD inhibition can give rise to neoantigenic peptides detected by the immune system. Moreover, the tumor cellular landscape of NMD-escaping frameshifted transcripts can be used as a biomarker of value for personalized immunotherapy design and prediction of its response

As above referred, some cancer types would benefit from a therapy based on NMD activation. This could be achieved by NMD-activating small molecules, with clinical utility to treat cancers in which the tumor-suppressive functions of NMD are beneficial, including solid tumors adapted to their own stressful microenvironment (Fig. 4a) [2, 8]. Despite being a simple approach in theory, this type of compounds may be difficult to develop. An easier alternative would be the development of oligonucleotides designed to re-engage NMD of cancer- and/or stress-related transcripts (Fig. 4a) [2]. In line with this, Tano et al. reported that treatment of human breast cancer cells (MCF10A and MCF10CA1h) with an NMD-inducing Morpholino against the signal transducer and activator of transcription 3 (STAT3) significantly reduces expression of this oncogenic transcription factor [193]. Despite this treatment had no effect on aspects of tumor cell biology (cell growth, viability, migration, and invasion) in the studied models, it highlights the therapeutic potential of using oligonucleotides to promote NMD of specific transcripts. This approach, however, would not be enough for tumors with affected expression of NMD factors, like ASC, ADCs and HCCs. In these cases, gene therapy aiming to induce expression of functional UPF1 could be of therapeutic value. To our knowledge, there are no studies so far directly testing this approach in the context of cancer. However, there are promising results in other diseases, like neurodegeneration. It was reported that transduced expression of human UPF1 in rat models of motor paralysis ameliorates the disease phenotype promoted by transactive response DNA-binding protein 43 kDa (TDP-43) [194]. Thus, the same results could be expected when applying this therapy to cancer models. Accordingly, gastric cancer cells (BGC-823 and SGC-7901) stably overexpressing UPF1 exhibit decreased cell proliferation, cell cycle progression, cell migration and invasion, and increased apoptosis, when compared to the control counterparts [195].

NMD activation therapy has the additional possibility of being used in combination with chemotherapy to protect normal cells from apoptosis [8]. This comes from the finding that chemotherapeutics promote NMD inhibition through caspase-mediated cleavage of UPF1 and UPF2, which creates truncated fragments with dominant-negative activity that leads to the upregulation of apoptosis-related NMD targets [196, 197]. Therefore, if it comes to be proven that NMD activation therapy increases normal cell survival, it will be possible to administer higher doses of chemotherapeutics to cancer patients to facilitate recovery after chemotherapy sessions [8]. Moreover, Li et al. reported that enforced expression of UPF1 can enhance sensitivity of BGC-823 and SGC-7901 cells to the chemotherapeutic, doxorubicin [195]. Thus, this therapeutic approach can potentially be used to increase the efficiency of chemotherapy itself.

Although NMD activation has great potential to be used in cancer therapy, the suggested approaches and methods are less developed than NMD inhibitory strategies. Therefore, there may be several years before being implemented in clinical practice.

### Global and gene-specific NMD inhibition in cancer therapy

Nonsense suppressors represent a class of compounds that enhance the readthrough of PTCs by the translating ribosome [158]. Given this mechanism of action, these compounds can “silence” the quality control facet of NMD and promote the production of a full-length protein, providing interesting therapeutic applications (Fig. 4b). In fact, nonsense suppressors have already been tested not only in the context of cancer, but in other genetic diseases, like cystic fibrosis and Duchenne muscular dystrophy, with positive outcomes [198–200]. Specifically in cancer, readthrough therapy can induce the production of protective tumor suppressor proteins encoded by PTC-harboring transcripts, as was described for p53 [198]. Furthermore, in the discussed case of CRCs with MSI, it was reported that NMD inhibition with amlexanox, a presumed nonsense suppressor, reduces cell proliferation in MSI CRC HCT116 cells and has an antitumor effect on MSI tumor xenografts, possibly being an effective strategy for the personalized treatment of CRC with MSI [157]. Alternatively, there is a plethora of other molecules that can inhibit NMD in different ways and that could be used for cancer therapy. This includes translational inhibitors (that therefore will inhibit NMD), small inhibiting molecules, such as NMD inhibitor 1 (NMDI-1) that stabilizes hyperphosphorylated UPF1 and reduces the UPF1-SMG5 interactions [201], cardiac glycosides that increase cytoplasmic calcium [reported to inhibit NMD [202]], and cytoskeleton disrupting agents, which interfere with the transportation process of PTC-containing transcripts for degradation [203]. However, in most cases, this approach is only effective when combined with readthrough therapy, as most of these drugs would simply promote the stabilization of nonsense-mutated transcripts, not inducing production of the correspondent functional, full-length proteins. Accordingly, Martin et al. have identified several compounds capable of interfering with SMG7-UPF1 interaction that, when used in combination with a nonsense suppressor, could restore p53 full-length protein and consequently promote the upregulation of p53 downstream transcripts, and cell death [204].

The usage of small inhibiting molecules/drugs to decrease NMD activity may present, however, a potential problem regarding their specificity, as these compounds might have off-targets that can compromise other cellular pathways or processes. This is aggravated by the fact that NMD targets several non-mutated transcripts, thus making it difficult to find the therapeutic dose of a global NMD inhibitor without having a major impact on the transcriptome. To overcome these issues, siRNAs and chemically modified antisense oligonucleotides (ASOs) designed to target NMD factors could provide a more specific therapeutic approach (Fig. 4b) [158]. Targeting NMD-substrates would be an even better alternative, because this would be expected to affect NMD of a single transcript. Accordingly, using ASOs to specifically block the deposition of an EJC downstream of a PTC could preclude its recognition by the NMD machinery. A practical example for this approach was recently provided by Rahman et al., who have showed, in HeLa cells, that the gene-specific blocking of EJC deposition using ASOs could avoid aberrant NMD of PTC-containing transcripts promoted by dysregulated AS events [159]. Nevertheless, once again, this type of therapy would only promote the stabilization of the PTC-containing mRNA, which could still encode a dominant-negative or non-functional protein. Therefore, this method would need to be combined with a readthrough compound to ultimately produce a functional full-length protein [158]. In line with this, Nomakuchi et al. have showed in vitro that the ASO-mediated NMD inhibition of a well-characterized NMD-substrate, the β-globin (HBB) gene with a nonsense mutation on codon 39, used in combination with the readthrough compound, G418, could restore expression of the full-length HBB protein [205]. Thus, the gene-specific NMD inhibition combined with readthrough therapy represent a promising methodology to tackle tumors expressing dominant-negative or non-functional forms of tumor-suppressor proteins.

### Global NMD inhibition or escaping in cancer immunotherapy

Early studies during the 90s in murine models revealed that T lymphocytes (or their effector molecules) are determinant to tumor control [206–208], suggesting a relationship between the organism’s immune system and cancer. Very soon this idea was also observed in humans, in which clinical data supported a role for T lymphocytes in tumor immune surveillance. Accordingly, spontaneous regression of malignant melanomas was observed in the context of vitiligo (an autoimmune disease), and has been correlated with the presence of tumor-associated CD4+, activated T lymphocytes [209], and with an immune response to the tumor-associated antigen, melan-A/MART-1, mediated by specific cytotoxic lymphocytes [210]. This interaction between cancer and the immune system can be easily understood considering the highly mutagenic nature of many tumors: as mutations accumulate within cancer cells, several indels lead to the production of frameshifted transcripts encoding proteins that can act as neoantigens and trigger an immune response against the tumor [211]. In contrast, cancer cells have also exploited the natural immune checkpoint mechanisms (which normally act as fail-safes to prevent aberrant activation of the immune system and maintain immune homeostasis) to dampen anti-tumor T cell responses and prevent their immunity-mediated destruction [212]. This includes the expression of CD80/CD86 and PD-L1 ligands by the tumor [or the tumor-associated antigen-presenting cells (APCs)] that interact with the negative regulators of T cell activation, the CTLA-4 and PD-1 receptors, respectively, expressed at the membrane of the activated immune cells [reviewed in [212, 213]]. A better comprehension of the stimulatory and co-inhibitory immune mechanisms in the past few decades provided an opportunity to develop a new therapeutic approach to cancer, the immunotherapy. Cancer immunotherapy consists in the usage of immune checkpoint inhibitors that target CTLA-4, PD-1, and PD-L1, to activate or restore the immune system and counteract the resistance of cancer cells to the immune responses (Fig. 4c) [212]. The promising results obtained with these immune checkpoint inhibitors in cancer patients led the U.S. FDA to approve several drugs for numerous cancer treatments in recent years, as reviewed in [212].

Despite the therapeutic advantages provided by the immune checkpoint blockade therapy in cancer, its efficacy is highly variable and the response rates are usually below 40% [212, 214, 215]. An important limitation might be the lack of a pre-existing anti-tumor immune response on which the checkpoint inhibitors can act [212]. In agreement with this, tumor biopsies revealed that melanoma regression following PD-1 blockade requires pre-existing CD8+, tumor-infiltrating T cells [216]. Similarly, an immune-active tumor microenvironment was reported to mediate the antitumor activity of CTLA-4 blockade [217]. Altogether, this stresses out the need to develop complementary therapeutic approaches to stimulate tumor-specific T cell responses and, thus, increase the efficacy of cancer immunotherapy.

As already mentioned, a key marker for immunotherapy response is the overall mutation burden of the tumor, i.e. the higher the number of accumulated frameshifting mutations, the greater the propensity of the tumor to produce neoantigenic peptides/proteins that can be detected by the immune system of the individual (Fig. 4c) [218]. Given that frameshift mutations usually result in PTC acquisition, Lindeboom et al. have recently hypothesized that NMD could also be involved in the efficacy of cancer immunotherapy [219]. Under lower pressure of the NMD pathway over PTC-harboring transcripts, there are more chances of producing neoantigens and therefore, higher immune infiltration. Indeed, after analysis of a pan-cancer cohort, the authors found that the burden of frameshift mutations that evade NMD correlates with increased tumor immune reactivity, suggesting a better response to immunotherapy [219]. Accordingly, Pastor and colleagues reported that the targeted inhibition of NMD in tumor cells leads to the expression of new antigens that are recognized and rejected by the immune system [220]. In fact, they showed that NMD inhibition in subcutaneous and metastatic tumor models leads to a significant reduction in tumor growth due to an enhanced tumor antigenicity. Therefore, NMD impairment, and the consequent preservation of PTC-harboring transcripts, seem to enhance the expression of neoantigens and the tumor response to immunotherapy [219, 220]. Given this, the described global NMD inhibitors have the potential to be used in a clinical context, either isolated or in combination with immune checkpoint inhibitors, for cancer immunotherapy [219]. These findings raised the possibility that the number of NMD-escaping frameshifting indels could be used as a biomarker of immunotherapy response (Fig. 4c), in addition to the tumor mutation burden. Accordingly, Litchfield et al. showed that NMD-escaping frameshift mutated transcripts could help to predict response to immunotherapy in patients with low tumor mutation burden [221]. Moreover, in the study of Lindeboom et al., a model for classification of patients as responders to immunotherapy based on the tumor mutation burden and the NMD-escape frameshift mutation number provided a 5.86% increase in sensitivity over a model based only on the tumor mutation burden [219]. In the same line, very recent data from the combination of long-read and short-read cDNA sequencing have provided a catalog of full-length transcript species in a series of lung cancer cells. Based on this catalog, and considering the encoded full-length protein sequences, authors also identified potential neoantigens that can serve as predictive markers for immunotherapy [222]. Together, these data is of major importance, as not only NMD inhibition may be an effective strategy to improve responsiveness to cancer immunotherapy, but also the landscape of tumor frameshifted transcripts that escape NMD can be used to design personalized immunotherapy and predict its response.

## Conclusions

The relationship between NMD and cancer is a complex one, where both its quality-control and regulatory functions can contribute to different stages of tumor development. Under normal circumstances, NMD is expected to have a protective role in the cell, preventing production of potential dominant-negative proteins and downregulating the expression of many important factors that can promote tumorigenesis [2, 26, 28, 107, 146–150]. However, growing evidence strongly suggests tumor cells may also be able to take advantage of such NMD functions to promote disease onset and progression. Indeed, one might view the role of NMD in cancer as a double-sided coin as it can act both as a tumor suppressor and a tumor promoter pathway depending on the genetic context of the cancer. Therefore, it seems of utmost importance to further explore the relevance of NMD in different cancer types and its exact mechanisms as this may set the ground for the development of novel therapeutic strategies. The development of such therapeutic strategies based on NMD modulation represents a challenge not only due to the complexity and specificity of the NMD-cancer relationship, but also due to the complexity of the disease itself. In addition, very recent data has revealed the potential of tumor frameshifted transcripts that escape NMD to encode neoatigens that can elicit immune response and function as markers for cancer immunotherapy design and prediction of its response.

Thus, it should be preconized the long-range sequencing of the tumor full-length transcriptome and the rules and effects of NMD should be incorporated into clinical decision-making processes, as, together, this information may better support personalized immunotherapy design.

## Data Availability

Not applicable.

## Abbreviations

aa: Amino acid
ADCs: Lung adenocarcinomas
AML: Acute myeloid leukemia
APCs: Antigen presenting cells
AS: Alternative splicing
ASC: Adenosquamous carcinoma
ASOs: Antisense oligonucleotides
ATF: Activating transcription factor
BCL-X: B-cell lymphoma extra
BRCA: Breast cancer susceptibility protein
CCR4-NOT: Deadenylation complex
CDH1: Cadherin 1
CRC: Colorectal cancer
CTLA-4: Cytotoxic T lymphocyte associated antigen 4
CYR61: Cysteine-rich angiogenic inducer 61
DCPC: Decapping complex
CHOP: CCAAT-enhancer-binding protein homologous protein
DECID: Decay-inducing complex
DHX34: DEAH box polypeptide 34
eIF: Eukaryotic initiation factor
EJC: Exon junction complex
EMT: Epithelial-mesenchymal transition
ER: Endoplasmic reticulum
eRF: Eukaryotic release factor
FDA: Food and Drug Administration
GADD45: Growth arrest and DNA damage 45
GAS5: Growth arrest specific 5
GLI1: Glioma oncogene homolog 1
GSH: Glutathione
HBB: Beta-globin
HCCs: Hepatocellular carcinomas
HDGC: Hereditary diffuse gastric cancers
HER2: Human epidermal growth factor receptor 2
hnRNPs: Heterogeneous nuclear ribonucleoproteins
IMTs: Inflammatory myofibroblastic tumors
ISR: Integrated stress response
KHDRBS1: KH RNA binding domain containing, signal transduction associated 1
MAP 3 K14: Mitogen activated protein kinase kinase kinase 14
MART-1: Melanoma antigen recognized by T cells 1
METTL3: Methyltransferase like 3
MSI: Microsatellite instability
mRNA: Messenger RNA
NCOR2: Nuclear receptor corepressor 2
NF-κB: Nuclear factor-kappa B
NMD: Nonsense-mediated RNA decay
NMDI-1: NMD inhibitor 1
nts: Nucleotides
PABPC1: Cytoplasmic poly(A)-binding protein 1
PAX3: Paired box gene 3
PC3: Prostate cancer
PD-1: Programmed cell death-1
PD-L1: Programmed death-ligand 1
PNRC2: Proline rich nuclear receptor coactivator 2
PP2A: Protein phosphatase 2A
PTBP1: Polypyrimidine tract-binding protein 1
PTCs: Premature termination codons
RB1: Retinoblastoma 1
RBP: RNA-binding protein
ROS: Reactive oxygen species
RSE: Stability element
SFV: Semliki forest virus
SINV: Sindbis virus
SLC7A11: Solute carrier family 7 member 11
SMD: Staufen-mediated decay
SR: Serine and arginine-rich
SRSF: Serine and arginine rich splicing factor
STAT3: Signal transducer and activator of transcription 3
TDP43: TAR (transactive response) DNA-binding protein 43
TGF-β: Transforming growth factor beta
tGLI1: Truncated GLI1
uORF: Upstream open reading frames
UPF: Up-frameshift
UPR: Unfolded protein response
UTR: Untranslated region
WT1: Wilms’ tumor protein 1
WT: Wild type
